# Gemtuzumab-Ozogamicin-Related Impaired Hemoglobin-Haptoglobin Scavenging as On-Target/Off-Tumor Toxicity of Anti-CD33 AML Therapy: A Report of Two Cases

**DOI:** 10.1155/2021/6641349

**Published:** 2021-03-20

**Authors:** Hanna L. M. Rajala, Veli-Jukka Anttila, Mikko Haapio, Mikko A. I. Keränen, Ulla Wartiovaara-Kautto, Riikka Räty

**Affiliations:** ^1^Department of Hematology, Helsinki University Hospital Comprehensive Cancer Center and University of Helsinki, Helsinki, Finland; ^2^Division of Infectious Diseases, Inflammation Center, Helsinki University Hospital and University of Helsinki, Helsinki, Finland; ^3^Nephrology, Helsinki University Hospital and University of Helsinki, Helsinki, Finland; ^4^Applied Tumor Genomics Research Program, Faculty of Medicine, University of Helsinki, Helsinki, Finland

## Abstract

Gemtuzumab-ozogamicin (GO) is a humanized anti-CD33 antibody, which is conjugated to a cytotoxic calicheamicin. It is used to treat acute myeloid leukemia (AML) in combination with chemotherapy. We describe here two GO-treated acute myeloid leukemia (AML) cases: both patients suffered from a toxic syndrome, which manifested as impaired hemoglobin-haptoglobin scavenging and accumulation of hemolysis-related products. Our observations and earlier reports indicated that the reaction was caused by GO-targeted destruction of CD33 + CD163+ monocytes/macrophages, which are responsible for the clearance of hemoglobin-haptoglobin complexes. The rise of plasma lactate dehydrogenase was an early sign of the reaction, and both patients had high levels of free plasma hemoglobin, but plasma haptoglobin and bilirubin levels were paradoxically normal. Symptoms included septic fever and abnormalities in cardiac tests and in the case of the first patient, severe neurological symptoms which required intensive care unit admittance. Therapeutic plasma exchanges supported the patients until the recovery of normal hematopoiesis. The symptoms may be easily confounded with infectious complications-related organ damage. Regarding the increasing use of gemtuzumab-ozogamicin and other emerging CD33-targeted cell therapies, we want to highlight this mostly unknown and probably underdiagnosed toxicity.

## 1. Introduction

Gemtuzumab-ozogamicin (GO) is a humanized anti-CD33 monoclonal antibody, which is linked to a very potent cytotoxic agent and N-acetyl gamma calicheamicin. CD33 is expressed on the surface of most acute myeloid leukemia (AML) blasts and on immature myeloid cells. [1] Adverse events related to GO, such as cytopenias and liver toxicity, led to its withdrawal from the market in 2010 due to failure to demonstrate survival benefit because of increased toxicity-related mortality. [2] In 2017, GO regained its approval from the FDA and EMA, as studies on lower-dose fractionated GO showed a better safety profile and beneficial results overall, especially in AML patients with favorable cytogenetics [3–6].

In this report, we present two patients who suffered from life-threatening GO-related impaired hemoglobin-haptoglobin scavenging, which manifested as an atypical intravascular hemolytic reaction. Earlier anecdotal reports have suggested that GO-targeted destruction of CD33 + CD163 + monocytes/macrophages leads to abrogated clearance of hemolysis-related products [7, 8]. Regarding the increasing use of GO and other emerging CD33-targeted cell therapies, we want to highlight this unknown and poorly defined on-target/off-tumor toxicity and share our experience of successful salvage with therapeutic plasma exchanges (TPEs). Both patients consented to the publication of the clinical data.

## 2. Case Presentation

**Figure 1:**
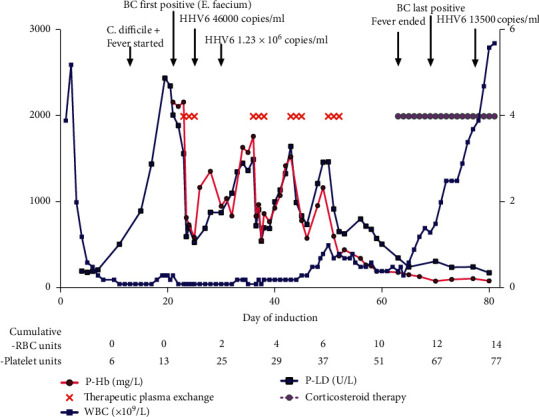
The clinical course of patient 1 (index case). Free plasma hemoglobin (P-Hb) and plasma lactate dehydrogenase (P-LD) are shown on the left y-axis, and white blood cell count (WBC) on the right *y*-axis. We used metronidazole and fidaxomicin to treat colitis caused by *Clostridium difficile* and valaciclovir for oral herpes simplex virus (HSV) infection. On day 21, blood culture (BC) was positive as the patient had *Enterococcus faecium* septicemia which continued the following weeks despite repeated removals and replacements of catheters and use of in vitro-effective antibiotics (tigecycline, linezolid, gentamicin, and daptomycin, which were selected because the patient was allergic to vancomycin). The patient tested positive for human herpes virus 6 nucleic acid test (HHV6-NAT) on day 25 and foscarnet was administered from day 31. Therapeutic plasma exchanges started on day 23 with transient responses to P-Hb and P-LD levels. The first signs of granulopoiesis were seen in bone marrow (BM) aspirate on day 46, and we were able to withhold TPEs after day 52, as the reaction subsided along with recovering hematopoiesis. Cumulative number of blood products transfused is shown below: there was no sign of hemolysis-related increased red blood cell (RBC) consumption, as we transfused the first RBC unit on day 25.

*Patient 1*: A 21-year-old otherwise healthy female was diagnosed with core-binding factor (CBF)-AML (t (8; 12) (q21.3; q22)), corresponding RUNX1/RUNX1T1 transcript. After the first-line therapies (Table 1), minimal residual disease (MRD) positivity of 0.02% was still detected in the bone marrow (BM). The patient then moved to Finland. At the first visit to our center, a florid BM-relapse was detected only 14 weeks after the start of the last consolidation cycle. We initiated a course of 7 + 3 combined with fractionated GO (Table 1). Along with stomatitis and cytarabine-related cutaneous reaction, from day 13 onwards, the patient suffered from high fever, malaise, *Clostridium difficile* colitis, and oral HSV infection. Moreover, *Enterococcus faecium* septicemia persisted from day 21, though no deep foci of infection or endocarditis were detected. Human herpes virus 6 nucleic acid test (HHV6-NAT) was positive on day 25 (Figure 1). As a first sign of a toxic reaction, plasma lactate dehydrogenase (P-LD) started to rise from day 11. The reaction escalated quickly from day 19, when blood samples became macroscopically hemolytic. Free plasma hemoglobin (P-Hb) rose markedly, but P-haptoglobin (P-Haptog) and P-Bilirubin (P-Bil) were paradoxically normal, unlike in typical intravascular hemolysis (Figure 1, Table 2). Kidney and liver tests remained normal, but plasma troponin I level was elevated and the patient was hypertensive.

To lower the amount of toxic P-Hb and other potentially harmful substances related to the hemolysis-like reaction and to prevent further organ damage, therapeutic plasma exchanges (TPEs) were performed on days 23–25, 36–38, 43–45, and 50–52. In each TPE, one volume of plasma (about 5% of the patient's weight) was exchanged and replaced with 4–5% albumin and Octaplas-LG® plasma via dialysis catheter. We used citrate and the low-molecular weight heparin, enoxaparin, as anticoagulants.

Despite wide-spectrum antibiotics and repeated TPEs, septic fever did not respond to therapy. On day 35, the patient was admitted to the intensive care unit (ICU) for four days after a seizure. MRI scan showed cerebral microhemorrhages. Cerebrospinal fluid (CSF) HHV6-NAT was positive.

High levels of P-Hb and P-LD persisted until the first signs of recovering hematopoiesis on day 45 (Figure 1, Table 2). However, fever continued and new viral pneumonia-like changes were seen in the CT scan with subacute cardiac tamponade requiring percutaneous pericardial drainage. Immunological pericarditis and pneumonitis were suspected and prednisolone 1 mg/kg was started on day 63 with a clinical response. The patient could finally be discharged on day 81.

During follow-up, cardiac function has recovered and pulmonary function improved partially. The patient remained in MRD-negative complete remission (CR) and underwent allogeneic stem cell transplantation.

*Patient 2*: a 24-year-old female was diagnosed with CBF-AML (inv16, corresponding *CBFB-MYH11* fusion gene). Pulmonary tuberculosis had been cured with nine months of four-drug therapy one and a half years ago, but otherwise her medical history was unremarkable. We initiated a course of 7 + 3 combined with a single dose of GO (Table 1). Wide-spread cytarabine-related cutaneous reaction and mucositis and high fever were observed from day 4 onwards. Blood cultures and virus samples remained negative. Because of suspected hyperinflammation (P-ferritin >16500 *μ*g/L, P-IL2R 5509 kU/L), we administered corticosteroid from day 16: fever responded but P-LD was rapidly increasing. P-Hb rose (410 mg/L at highest), and the color of patient's plasma slightly darkened while P-Haptog and P-Bil stayed normal. Hypertension and bradycardia were observed. We did not see any other sign of target-organ damage caused by the toxic reaction. We conducted preemptive TPEs daily on days 20–22. No more TPEs were needed, as P-LD and P-Hb declined after day 22 together with recovering hematopoiesis. CR with low MRD-positivity (*CBFB-MYH11* 0.02%) was detected in the BM on day 29.

## 3. Discussion

We describe here a rare but life-threatening GO-related on-target/off-tumor toxicity, from which the patients were salvaged with TPEs until hematopoietic recovery. Based on earlier publications, we suspected a hemolysis-like reaction and accumulation of hemolysis-related products caused by impaired hemoglobin‐haptoglobin scavenging [7, 8]. Previous reports describe patients who experienced septic high fever and overall malaise, hypertension, neurological symptoms, and abdominal pain. None survived, either due to the persistent reaction or progressive disease (Table 1).

In intravascular hemolysis, plasma haptoglobin is depleted as it binds excess free hemoglobin to prevent its toxicity and infiltration by the kidneys. CD33+ mature tissue macrophages internalize the complexes via the CD163 scavenger receptor [9]. Maniecki et al. speculated that anti-CD33-mediated destruction of CD33 + CD163+ macrophages and subsequently impaired CD163-mediated hemoglobin-haptoglobin scavenging leads to the paradoxical accumulation of both plasma hemoglobin and haptoglobin [7]. Our two patients did not have eminent intravascular hemolysis, as they did not require red blood cell transfusions at the beginning of the reaction. The origin of excess P-Hb is not known, but we suspect that it might be partly related to anti-CD33-mediated destruction of red pulp macrophages in the spleen: these cells are responsible for maintaining hemoglobin-haptoglobin scavenging, and their destruction might release free hemoglobin into plasma. [10].

TPE is used to remove antibodies and immune complexes from circulation [11]. It is the standard of care in thrombotic thrombocytopenic purpura, in which it removes ADAMTS13-blocking antibodies [12]. However, TPE is not usually effective in autoimmune hemolytic anemia [13]. In our patients, we used TPE to prevent toxicity related to free plasma hemoglobin, heme, and other potentially toxic molecules related to an inflammatory condition.

The incidence of impaired hemoglobin-haptoglobin scavenging related to GO is probably higher than reported, and the clinical presentation may vary from asymptomatic to life-threatening. Infectious complications and related organ damage during AML therapy may confound or delay the detection of the reaction. The currently recommended lower dose of GO seems to be better tolerated, but the reaction is probably dose-independent. Other CD33-targeting therapies, such as CD33-directed chimeric antigen receptor (CAR) T cells, are under investigation [14]. Similar toxicity seems possible during CD33 CAR T cell therapy, and one might speculate that the reaction could even be prolonged due to the persistence of CAR T cells and enhanced by cytokine release syndrome.

The trigger responsible for tilting the scales towards P-Hb accumulation is not known. Patient 1 had severe infectious problems and patient 2 suffered from widespread cytarabine-related skin toxicity, which might have contributed to the hyperinflammatory status. We did not detect germline mutations linked to immune deficiencies or increased susceptibility to toxicity (skin biopsies and gene panel sequencing).

According to our experiences, P-LD seems to be an early biomarker for this severe adverse event. We suggest that P-LD is followed in GO-treated patients, and if it rises, especially 1–2 weeks after GO administration (outside the time frame for tumor lysis), P-Hb should be analyzed as well. Our first patient survived this toxicity with repeated TPEs, and a similar reaction was suspected in the case of the second patient. Based on our experience, the initiation of TPEs might support patients through bone marrow hypoplasia, until the recovery of normal hemoglobin-haptoglobin scavenging.

## Figures and Tables

**Table 1:** Summary of the five patients who had impaired hemoglobin‐haptoglobin scavenging reaction after gemtuzumab-ozogamicin therapy. The first two cases are described in detail in this paper, and the last three patients are summarized from two previous publications. In addition, Maniecki et al. [7] mentioned one adult patient, from whom clinical details were not reported.

Patient	Diagnosis	Frontline therapy	Therapy of relapse	Time from last GO to hemolysis	Infections	Symptoms during hemolysis	Outcome	Publication
Patient 1 : 21-year-old female (index patient)	Relapsed CBF-AML (BM-blasts 65%) *t* (8; 12), corresponding *RUNX1/RUNX1T1*-fusion gene. Additional mutations in *PHF6*, *EZH2*, and *KDM6A.* No extramedullary disease.	7 + 3 (cytarabine + daunorubicin), 3 x HD-AraC. Relapse after 2 mo.	7 + 3 (cytarabine and idarubicin) and **GO (3 mg/m^2^, days 1, 4, 7)**	19 days	*Enterococcus faecium* sepsis, *Clostridium difficile* colitis, HHV6 viremia, HSV stomatitis	Fever, convulsions, confusion, mild diastolic hypertension	Recovered after repeated TPEs. Response to therapy: CRi, MRD-neg	Current
Patient 2 : 24-year-old female	Primary CBF-AML (BM-blasts 69%) Inv16, corresponding *CBFB-MYH11*-fusion gene. Axillary and inguinal lymphadenopathy.	7 + 3 (cytarabine and idarubicin) and **GO (3 mg/m2 on day 4)**	—	14 days	Suspected infection, blood cultures negative and virus samples negative. No deep foci of infection detected.	Fever, hypertension, bradycardia	Recovered after 3 TPEs. Response to therapy: CR, MRD 0.02%	Current
2-year-old boy	Relapsed AML M4/M5	NOPHO-AML93 + MUD-SCT. Relapse 18 mo after SCT.	FLAG and **2 doses of GO (7.5 mg/m2) at a 2-week interval**	3 weeks	*Staphylococcal* sepsis	Abdominal pain, hypertension, persistent high fever, malaise	Recovery, but died later from PD	Maniecki et al. Blood 2000 [7]
1-year-old girl	Relapsed AML M5	NOPHO-AML 2004 with 2 doses of GO (5 mg/m^2^, 2-week interval). Relapse after 3 mo.	FLAG-DaunoXome, **2 doses of GO (6 mg/m^2^, 2-week interval)** and NOPHO-AML 2004 induction	5 weeks	Suspected infection, blood cultures negative	Hypertension, confusion, convulsions	Did not recover from hemolytic episode and died from PD 72 days from the first GO	Maniecki et al. Blood 2000 [7]
12-year-old boy	Refractory AML	Relapse after two salvage chemotherapies	**GO on days 1 and 15**, dose not reported	Hemolytic samples from day 5 of GO cycle	Vancomycin-resistant *Enterococcus faecium* and *Trichosporon asahi*i infections	Hypertension, multiorgan failure and need for renal replacement therapy	Did not recover from hemolysis and died 40 days after the first GO	Tesfazghi et al. Clin Chem 2018 [8]

*Abbreviations*. AML: acute myeloid leukemia; BM, bone marrow; CBF, core-binding factor; CR, complete remission; CRi, CR with incomplete recovery of blood counts; FLAG, fludarabine-HD-AraC-granulocyte stimulating factor; GO, gemtuzumab-ozogamicin; HD-AraC, high-dose cytarabine; HHV6, human herpes virus 6; HSV, herpes simplex virus; mo, months; MRD, minimal residual disease; MUD, matched unrelated donor; NA, not accessible; PD, progressive disease; SCT, stem cell transplant; and TPE, therapeutic plasma exchange.

**Table 2:** Patient 1: laboratory findings and cumulative number of transfused blood products at different timepoints.

Test unit (normal range)	Day 1 of induction	Day 11 (P-LD rising)	Day 23 (before 1^st^ plasma exchange)	Day 52 (last plasma exchange)	Day 63 (cardiac tamponade)	Day 81 (discharge)	Day 98 (the last BM sample)
B-Hb g/dL (11.7–15.5)	11.9	12.1	NAA	7.4	7.5	9.3	9.9
B-WBC x10^9^/L (3.4–8.2)	3.9	0.1	0.1	0.8	0.4	5.7	6.0
B-PLTs x10^9^/L (150–360)	83	44	122	14	18	67	29
B-neutrophils x10^9^/L (1.5–6.7)	0.39	<0.05	<0.05	0.43	0.14	4.73	4.31
P-CRP mg/L (<4)	<4	28	411	216	266	57	33
P-Hb mg/L (<50)			2167	376	186	87	77
P-LD U/L (115–235)	201	513	1566	660	353	181	175
P-Haptog g/L (0.29–2.0)			4.53	1.46	0.61		
P-Ferritin *μ*g/L (15–125)	11		>16500	1609	1016	1918	2303
P-creatinine *μ*mol/L (50–90)	45	40	49	21	22	29	36
P-ALT U/L (<35)	<9	20	52	42	13	58	31
P-bilirubin *μ*mol/L (<20)	7	5	5	7	29	14	11
P-TnI mg/L (<45)	10		68	136	99	45	18
Cumulative number of RBC units	0	0	0	7	12	14	14
Cumulative number of PLT units	0	7	17	39	57	71	77

Abbreviations: ALT, alanine transferase; B, blood; BM, bone marrow; CRP, C-reactive protein; Hb, hemoglobin; LD, lactate dehydrogenase; NAA, not able to analyze; P, plasma; PLTs, platelets; RBC, red blood cell; TnI, troponine I; WBC, white blood cell.

## Data Availability

No data were used to support this study.
